# Exploring the Link Between the Gut Microbiota and Epigenetic Factors in Anorexia Nervosa

**DOI:** 10.1002/brb3.70733

**Published:** 2025-08-21

**Authors:** N. M. Korten, A. C. Thelen, C. Voelz, C. Beyer, J. Seitz, S. Trinh, L. Blischke

**Affiliations:** ^1^ Institute of Neuroanatomy Uniklinik RWTH Aachen Aachen Germany; ^2^ West German Center For Child and Adolescent Health (WZKJ) University Hospital Cologne Cologne Germany; ^3^ Institute of Functional and Applied Anatomy Hannover Medical School (MHH) Hannover Germany; ^4^ Department of Child and Adolescent Psychiatry, Psychosomatics and Psychotherapy LVR University Hospital Essen Essen Germany

**Keywords:** anorexia nervosa (AN), depression, DNA methylation, eating disorder, epigenetic, histone modification, mental disorder, microbiome, noncoding RNA, obesity

## Abstract

**Objective:**

Anorexia nervosa (AN) is an often chronic eating disorder that involves genetic, neurohormonal, and epigenetic factors along with key contributions from the microbiota–gut–brain axis. However, interactions between these factors are poorly understood. Recent studies have emphasized the microbiota–gut–brain axis and epigenetic changes as potentially important contributors to AN. Exploring these interactions may improve understanding of the etiology and persistence of AN.

**Methods:**

Studies specifically addressing microbial–epigenetic interactions in AN remain limited. However, similar associations have been documented in related disorders such as obesity and depression, providing potential models for AN research.

**Results:**

Research in obesity has shown that dietary factors influence the composition of the gut microbiota and subsequent epigenetic modifications, affecting metabolic parameters and disease progression. Similarly, in depression, microbially produced metabolites influence brain function and epigenetic processes, contributing to neuropsychiatric symptoms. In AN, altered microbial composition may affect weight regulation and epigenetic patterns. Therapies targeting the microbiome, such as fecal microbiota transplantation, are under investigation for AN, highlighting the potential therapeutic utility of ameliorating microbial dysbiosis.

**Discussion:**

This article highlights the importance of investigating microbial–epigenetic interactions in AN. By drawing parallels with obesity and depression, we aim to deepen our understanding of AN mechanisms and ultimately improve patient outcomes.

## Graphical abstract

Current knowledge about the complex causes and treatment approaches for anorexia nervosa is limited. While research has separately associated microbiome changes and epigenetic modifications with AN, the combined effects of these factors are not well understood, and exploring their interaction could lead to novel biomarkers and therapeutic options.

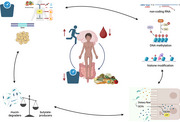

## 1 Introduction

Anorexia nervosa (AN) is an often chronic eating disorder (Herpertz et al. 2018; Watson et al. 2019) with a strong genetic component that manifests as reduced food intake and body weight, altered body perception, and fear of weight gain. Although the number of hospital admissions for AN has increased in recent years, the understanding of its complex pathophysiology remains elusive (D et al. 2023; Skowron et al. 2020).

Currently, the bidirectional link between the gastrointestinal tract and the central nervous system, known as the “microbiota–gut–brain axis,” has attracted increased research interest in metabolic and psychiatric diseases such as AN (Woo and Alenghat 2022; Andreani et al. 2024; Nohesara et al. 2023; Taniya et al. 2022; L. Zhang et al. 2020; M. Lee and Chang 2021). Furthermore, epigenetic factors (i.e., changes in gene activity that do not involve changes in DNA sequence (Steiger et al. 2023)) may alter the expression of genes associated with appetite, body weight, and mood regulation, adding another layer of complexity to the etiology and maintenance of AN (Remely and Haslberger 2017; Remely et al. 2015; Cuevas‐Sierra et al. 2019).

**FIGURE 1:**
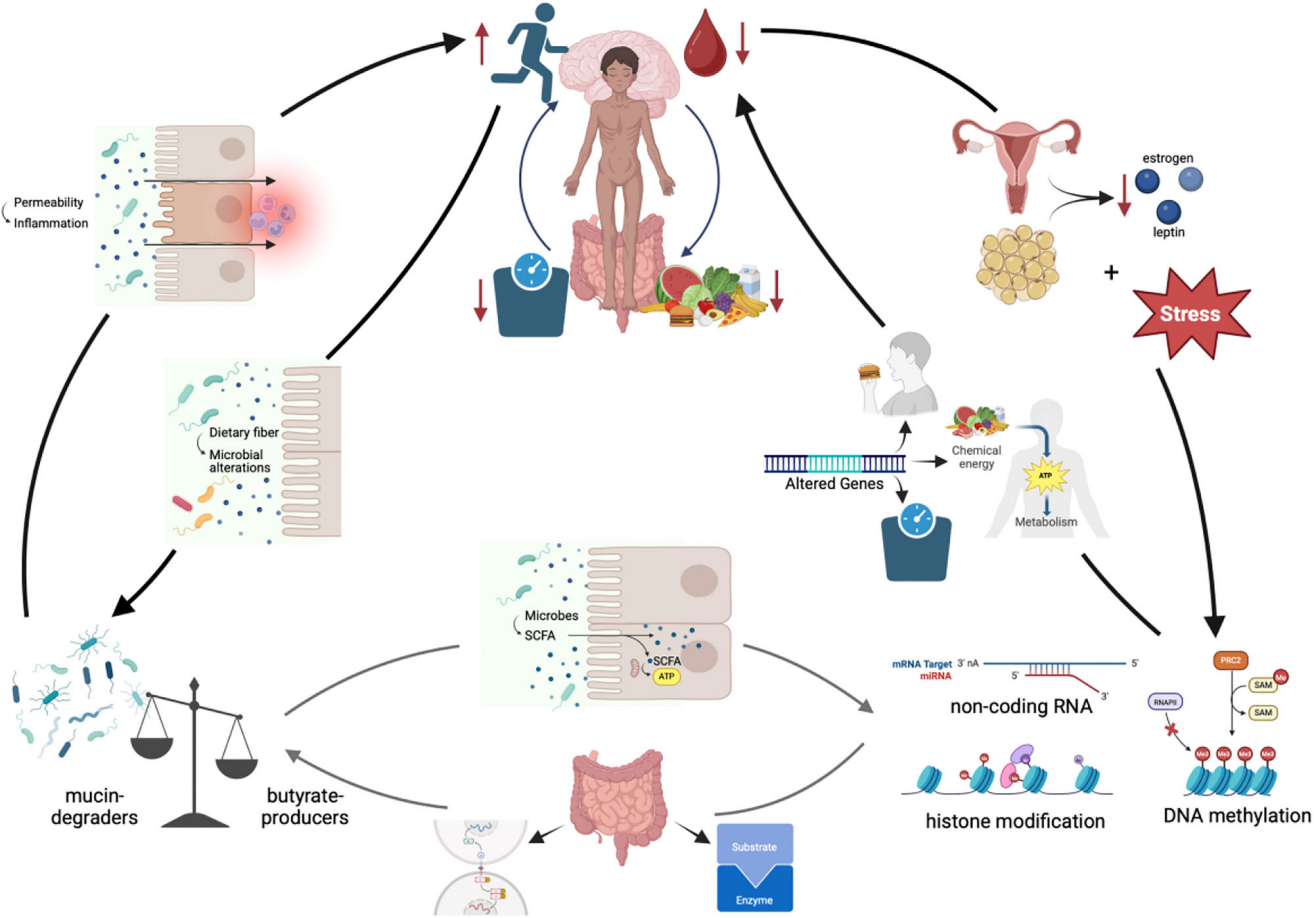
Schematic illustration of the interplay between epigenetics and the microbiome in the pathogenesis of anorexia nervosa. Previous research suggests that clinical symptoms of AN (reduced food intake, urge to exercise, weight loss, and amenorrhoea) are bidirectionally connected to microbiome alterations and changes in epigenetic patterns (histone modification, DNA methylation, and noncoding RNA). Pathways are highlighted with black arrows as literature‐based. An additional bidirectional interaction between the microbiome and epigenetics is yet to be researched in AN but can be derived from other diseases (e.g., obesity and depression). Pathways are illustrated with gray arrows as speculative and yet to be proven. Created in BioRender. Voelz, C. (2025) https://BioRender.Com/c83x141

Microbial alterations and epigenetic shifts are independently associated with the pathophysiology of AN (see review on microbiome in AN (Seitz et al. 2020) and epigenetics in AN (Käver et al. 2024)). Investigating the interplay between these two mechanisms is of interest because it may elucidate complex disease‐driving pathways (e.g., feedback loops) and allow a more targeted and personalized therapeutic approach (Figure 1). As the microbial–epigenetic interplay has already been identified as a potential causal pathomechanism in other diseases, dissecting and comprehending this interplay in AN is of great scientific and clinical interest (H. S. Lee 2019).

After extensive literature research in October 2024 in two different scientific databases (PubMed, Web of Science) with the following key search terms: (#1 anorexia OR anorexia nervosa) AND (#2 epigenetic OR epigenetics OR methylation OR histone OR noncoding RNA) AND (#3 microbiome OR microbiota), only three articles on PubMed and seven articles on Web of Science were found that mentioned microbial and epigenetic changes, but had to be excluded as none addressed their interaction in the context of AN (Gorwood et al. 2016; Himmerich et al. 2019; Seidel et al. 2020; Trinh et al. 2021; Wagner‐Skacel et al. 2020; Keenan et al. 2015; Wu et al. 2023).

At the same time, microbial–epigenetic interactions have been studied in AN‐related diseases such as obesity and depression. Despite their contrasts, obesity and AN represent extreme nutritional states, both characterized by diet‐induced perturbations of the gut microbiome and energy balance. It is therefore plausible that analogous epigenetic and microbial pathways involving weight‐regulating and metabolic genes are affected, even if the specific alterations show different or opposing patterns (Käver et al. 2024; Remely et al. 2014). Furthermore, depression is a common comorbidity of AN, with a prevalence ranging from 36% to 80% (Calvo‐Rivera et al. 2022). Both disorders share similar pathophysiological pathways, including disturbances in the microbial and epigenetic regulation of stress, mood, and behavior via the gut–brain axis (Park et al. 2019; Chang et al. 2022; Bou Khalil et al. 2017). Therefore, insights from obesity and depression research can reveal mechanisms relevant to AN.

In summary, understanding the interaction between gut microbes and epigenetics in AN is essential because (Herpertz et al. 2018) AN is the most lethal psychiatric disorder, with relapse rates reaching up to 50% (Hoang et al. 2014; Khalsa et al. 2017; Watson et al. 2019) AN treatment options currently focus on weight recovery and psychotherapy rather than the causal pathophysiology (Bryson et al. 2024; Devoe et al. 2023). The understanding of AN pathophysiology is limited to individual aspects, while a framework that integrates these elements is lacking. Accordingly, this article highlights the importance of investigating the interplay between the gut microbiome and epigenetic factors in AN and also presents relevant findings in obesity and depression.

### 1.1 The Microbiome and Epigenetics in AN

The human microbiome consists of numerous bacteria, viruses, archaea, and fungi and, as such, represents a large source of environmental stimuli that influence host physiology and phenotype (Woo and Alenghat 2022). From the five most commonly addressed phyla, *Firmicutes* and *Bacteroidetes* form over 90% of the bacterial composition, being crucial, for example, for metabolic pathways and immune function (Qin et al. 2010). Consequently, microbes and their metabolites have been identified as important players in the development and maintenance of several diseases (Jandhyala et al. 2015; Hidalgo‐Cantabrana et al. 2017; Belkaid and Hand 2014). Despite the existence of numerous studies that have addressed microbial shifts in AN, a clear definition of a “typical AN microbiome” remains elusive. Multiple studies have found heterogeneously shifted α‐diversity (number/relative abundance of species) and β‐diversity (difference in species between multiple samples) in patients with AN compared with healthy controls (Seitz et al. 2019; Kleiman et al. 2015; Mörkl et al. 2017). Several bacterial genera have been identified as having already attracted attention in connection with other metabo‐psychiatric diseases and/or as playing a role in nutritional metabolism. These bacterial taxa (foremost mucin‐degraders and butyrate‐producers) appear to be associated with the development and maintenance of AN and could be partially associated with clinical and eating disorder‐typical parameters (Seitz et al. 2020; Zhu et al. 2023; Ruusunen et al. 2019). More specifically, butyrate‐producing genera, such as *Faecalibacterium* and *Roseburia*, appear to decrease in abundance in patients with AN, whereas the levels of mucin‐degrading species, such as *Methanobrevibacter smithii and Akkermansia*, appear to increase (Zhao et al. 2024; Nikolova et al. 2021; Garcia and Gutierrez 2023; Di Lodovico et al. 2021). While butyrate‐producers protect the intestinal barrier by metabolizing carbohydrates into butyrate, mucin‐degraders can damage the mucin barrier (Parrish et al. 2022) (Seitz et al. 2020; Geirnaert et al. 2017). Since starvation results in a lack of carbohydrate intake, a decrease in butyrate producers combined with an elevation of mucin degraders can negatively affect gut permeability and inflammation (Mack et al. 2016; Jésus et al. 2014). Following weight rehabilitation, microbiome dysbiosis appears to show partial improvement (Andreani et al. 2024; Mack et al. 2016; Schulz et al. 2021). However, there is a correlation between bacterial taxa and clinical parameters during acute AN, with the potential to predict rehospitalization after one year (Andreani et al. 2024; E et al. 2013).

Since conventional treatment strategies have yielded limited success, innovative methods that directly target microbial composition have been suggested (Starzomska et al. 2020). To date, few human and animal studies have used fecal microbiota transplantation (FMT) to research or treat AN (Fan et al. 2023; Prochazkova et al. 2019; de Clercq et al. 2019; Glenny et al. 2021). Animal studies have provided initial evidence supporting a causal relationship between the gut microbiome and disease pathology by demonstrating reduced weight gain after transplantation of stools from patients diagnosed with AN compared with healthy control stools (Fan et al. 2023; Hata et al. 2019). The bidirectional microbiota–gut–brain axis, consisting of multiple direct and indirect pathways (metabolic, endocrine, neural, and immunological), allows gut microorganisms to impact brain functions in neuropsychiatric disorders (Generoso et al. 2021). Exemplarily, neuronal signals are transmitted via the vagal nerve, while small molecules, such as short‐chain fatty acids (SCFAs) and immune mediators, are exchanged via the blood vessels and affect the enteric nervous system locally (Góralczyk‐Bińkowska et al. 2022). Emerging microbiome‐targeted therapies, such as FMT in Clostridium difficile infection (Minkoff et al. 2023) or microbiome modulation to ensure the efficacy of cancer therapy (Natarelli et al. 2024), provide hope that patients with AN, as well as those with other eating disorders, may also benefit in the future.

Epigenetic mechanisms combine environmental and internal factors with genetic regulation via DNA/histone modifications and noncoding RNAs (Käver et al. 2024). These modifications affect the transcription process and thus gene expression but do not alter the DNA sequence itself. Mechanistically, SCFAs, including butyrate, acetate, and propionate, are important players in epigenetic modifications. These compounds regulate the posttranslational modification(s) of proteins, thereby influencing their dynamic functions and properties (Woo and Alenghat 2022; Kopczyńska and Kowalczyk 2024). They also directly influence host cell signaling or indirectly modify chromatin structure by inhibiting epigenetic enzymes in the gut (Bhat and Kapila 2017). Indicative of their relevance in AN, SCFAs have been identified to affect appetite regulation by promoting the secretion of satiety hormones via receptor binding in enteroendocrine cells (Z. Li et al. 2018; Psichas et al. 2015). SCFAs resulted in different shifts in AN levels, mainly with a reduction in acetate, butyrate, or propionate (Zhu et al. 2023). A systematic review by Käver et al. (2024) summarized further epigenetic alterations in patients with AN. The authors proposed several differentially AN‐associated methylated CpG sites (a DNA region with a cytosine nucleotide followed by a guanine nucleotide) in patients with AN compared to controls in candidate genes responsible for neuronal communication, lipid metabolism, thermogenesis, and weight regulation. In addition, CpG methylation appears to affect the leptin pathway, resulting in disturbed appetite and energy balance (Steiger et al. 2023). However, past studies have focused heavily on DNA methylation (DNAm) and have shown low reproducibility (Käver et al. 2024).

In a longitudinal study on epigenetic differences between patients with acute AN and weight‐recovered remitted patients, Steiger et al. revealed that most epigenetic alterations in AN appear to be reversible upon (weight) recovery, especially affecting metabolic and inflammatory genes. These results suggest that malnutrition and microbial changes might be the driving forces of these variations (Steiger et al. 2019). This reversibility also suggests that epigenetics should not be considered a disease‐causing factor but rather a driver of disease development and progression. Epigenetic patterns may serve as diagnostic biomarkers for disease staging and prevention (Käver et al. 2024). Microbial alterations may affect epigenetic patterns and, therefore, represent a target for potential interventions (Figure 1).

### 1.2 Interaction Between Gut Microorganisms and Epigenetic Factors: Current Insights From AN‐Related Diseases

Three microbial–epigenetic pathways, in which microbes and bacteria‐produced metabolites influence intestinal biology and promote disease(s), have been revealed (Woo and Alenghat 2022, D. Li et al. 2022; Sharma et al. 2019) [see review Woo and Alenghat 2022]): first, shifts in the availability of chemical donors for DNAm or histone modification via bacteria‐produced metabolites; second, changes in enzymes involved in epigenetic patterns; and, third, alterations in host intrinsic pathways that affect epigenetic patterns (Woo and Alenghat 2022).

Contrary to the lack of research on these interactions in regard to AN, in the field of AN‐related diseases, more findings have been published already exhibiting crucial pathways that might be relevant for AN as well. Exemplarily, microbial profiling in obesity‐mimicking animal models and humans has revealed epigenetic pathways influenced by microbial changes (Remely et al. 2015, D. Li et al. 2022; Sharma et al. 2019; Shenderov and Midtvedt 2014; Kumar et al. 2014).

In depression, gut microbes and their metabolites appear to influence stress‐related responses and behavior of patients by inducing various epigenetic processes (Nohesara et al. 2023; see Begum et al. (2022) for a review).

### 1.3 Influence of the Gut Microbiome on DNAm

Genes are inactivated by the addition of a methyl group (─CH_3_) to cytosine bases by DNA methyltransferases, normally at CpG sites (L. Zhang et al. 2020; Holliday and Pugh 1975). Thus, DNAm can control the variability of genes and promoter regions, which are further regulated by bacteria‐produced metabolites and dietary ingredients (Miro‐Blanch and Yanes 2019). Bacterial metabolites (e.g., folate, vitamin B_12_, betaine, and choline) are involved in the synthesis of methyl donors for conversion to *S*‐adenosyl‐l‐methionine, known as 5‐methyltetrahydrofolate, which forms important substrates for DNA and histone modification (Crider et al. 2012). Changes in metabolites’ composition influencing the availability of *S*‐adenosyl‐l‐methionine therefore lead to epigenetic shifts (Woo and Alenghat 2022; Sharma et al. 2019). Genome‐wide analysis has revealed divergent methylation patterns that are correlated with the levels of *Bacteroidetes* and *Firmicutes* (Ramos‐Molina et al. 2019). In particular, genes involved in glucose and energy metabolism appear to be affected (Kumar et al. 2014).

Regarding obesity, the reduced abundance of *Faecalibacterium prausnitzii* in patients was correlated with hypomethylation of the free fatty acid receptor 3 (*FFAR3*) gene. Activated by the binding of SCFAs, FFAR3 plays a critical role in metabolic processes (Remely et al. 2014). Hypomethylation‐induced overexpression of FFAR3 might therefore promote metabolic pathways, forming a risk for obesity. After weight loss, the methylation of the promoter region recovers, strengthening the connection between methylation patterns and an obese phenotype (Remely et al. 2014).

In depression, DNA hypermethylation of numerous genes has been observed in animals with increased depression‐like behaviors (Buchenauer et al. 2023). Interestingly, tryptophan hydroxylase 2 (a key enzyme for the synthesis of serotonin (5‐HT) in the central nervous system) hypermethylation has been linked to reduced cerebral serotonin synthesis and depression (Kulikova and Kulikov 2019). Meanwhile, the abundance of tryptophan‐metabolizing microbes, *Alistipes*, and *Blautia* was elevated. In another clinical study involving patients with polycystic ovary syndrome, higher depression scores were associated with microbial alterations (29 distinct bacterial genera between the PCOS and healthy groups, especially increased *Escherichia* in the PCOS group) and DNA hypomethylation of the *FKBP5* gene (a mediator of inflammation and stress responses) (Nohesara et al. 2023; Ising et al. 2019).

As studies have found an altered global DNAm profile in blood samples from patients with AN compared to controls (Käver et al. 2024), a link to a loss of bacterial metabolites due to a reduced nutritional intake seems reasonable. Epigenetic processes are highly tissue‐specific. Differences in DNAm within organs could be explained by the different accessibility of host metabolites and stores depending on cell‐specific mechanisms. Regulation of weight‐controlling and metabolism‐associated genes might be contrarily impacted, as evidenced in obesity development.

### 1.4 Influence of the Gut Microbiome on Histone Structure

Histone proteins stabilize DNA by facilitating its organization into a highly compact structure. This is achieved through the process of DNA winding around histone octamers, forming a unit known as chromatin. Histone modifications, such as methylation, acetylation, phosphorylation, and ubiquitination, affect the accessibility of DNA, thereby influencing gene expression. Improved DNA accessibility has been achieved using histone acetyltransferases by reducing the electrostatic attraction between DNA and histones. In contrast, histone methylation by histone methyltransferases can activate or inactivate specific genes depending on the localization (promoter area/regulatory regions) and type of methylation (mono‐, di‐, or tri‐methylation). (L. Zhang et al. 2020; Bhat and Kapila 2017). Bacterially produced SCFAs affect chromatin structure by inhibiting histone deacetylases, which are responsible for removing acetylation from the chromatin structure. This inhibition increases gene expression and enhances adipocyte differentiation by inducing relaxation of the chromatin structure, thereby increasing the accessibility of DNA to transcriptional machinery (Woo and Alenghat 2022; Sharma et al. 2019; Kasubuchi et al. 2015, G. Li et al. 2014; Krautkramer et al. 2016). Changes in SCFA production are manifested by changes in metabolic parameters, such as body mass index, insulin sensitivity, body weight, and fat mass (Remely et al. 2015; Cuevas‐Sierra et al. 2019; Remely et al. 2015, D. Li et al. 2022; Sharma et al. 2019; Shenderov and Midtvedt 2014; Kumar et al. 2014). Krautkramer et al. (2016) reported that germ‐free animals raised under sterile conditions exhibit histone modifications in the liver, proximal colon, and white adipose tissue compared with conventionally raised controls. Interestingly, these dysbiosis‐induced shifts of the epigenetic patterns were aligned with those of the controls after SCFA supplementation (Krautkramer et al. 2016).

In obese mice, the histone demethylase Jhdm2a is involved in regulating metabolic gene expression and can affect weight control. Thus, via β‐adrenergic signaling disruption, mice lacking Jhdm2a exhibit obesity and hyperlipidemia (Tateishi et al. 2009).

SCFA levels, particularly butyrate, are decreased in depression, possibly due to a reduction in butyrate‐producing species, such as *Faecalibacterium* spp. (Jiang et al. 2015). Thus, the lack of SCFAs in depression leads to reduced transcriptional activation and may contribute to reduced neurotransmitter levels such as serotonin and GABA (Nohesara et al. 2023; Begum et al. 2022; Sun et al. 2013). Moreover, an animal model of depression indicated that repeated administration of butyrate could lead to decreased hippocampal microglial activation and depression‐like behavior due to the inhibition of histone deacetylation (Yamawaki et al. 2018; Liu et al. 2023).

As an acetyl donor, acetyl‐CoA is crucial for histone acetylation. In this context, cultured mammalian cells exhibit an increase in acetyl‐CoA after glucose stimulation, leading to enhanced acetylation processes (J. V. Lee et al. 2014). Glucose deprivation, as in acute starvation of AN, leads to the downregulation of adipocyte differentiation due to a diminished CoA‐dependent histone acetylation (Wellen et al. 2009).

Despite the fact that divergent histone modifications in pathways potentially relevant to AN, such as weight‐controlling genes (Tateishi et al. 2009), exist, research regarding crucial alterations of histone acetylation and methylation patterns in AN remains limited.

### 1.5 Influence of the Gut Microbiome on Noncoding RNAs

Finally, noncoding RNAs, such as long noncoding RNAs (lncRNAs) and microRNAs (miRNAs), indirectly or directly affect histone and DNA methyltransferases and thereby gene expression (L. Zhang et al. 2020; Bhat and Kapila 2017). LncRNAs influence histone methyltransferases or demethylases directly or by recruiting chromatin‐modifying complexes (X. Zhang et al. 2019). miRNAs indirectly inhibit gene expression by binding to 3′‐untranslated regions of messenger RNA, leading to its degradation or blocking translation into specific proteins (L. Zhang et al. 2020).

Both noncoding RNAs are important for maintaining metabolic homeostasis, whereas changes caused by microbiota dysbiosis can promote metabolic diseases (Celiker and Kalkan 2020; Devaux and Raoult 2018). Studies have reported divergent lncRNA expression in the gut tissue of mice in different microbial states (Dempsey et al. 2018; Liang et al. 2015). These lncRNAs interact with protein‐coding genes, form tissue‐specific networks, and affect enzyme expression (Dempsey et al. 2018). Similar effects have been reported for miRNAs (Dalmasso et al. 2011). Their potentially distinct roles in AN are discussed in detail by Voelz et al. (2024). Noncoding RNAs are influenced by nutrition and microbial composition, linking microbial changes to epigenetic influences (Vikram et al. 2016).

Specific miRNAs have been suggested to be relevant to the development of obesity (Sharma et al. 2019; Trajkovski et al. 2011; Dávalos et al. 2011; Virtue et al. 2019). The progression of obesity is affected by the regulation of white adipose tissue browning and intestinal microbiota homeostasis via miR‐204 (Kassan et al. 2022). In a shotgun analysis of fecal samples from subjects on different diets (vegans and vegetarians), Tarallo et al. (2022) reported a significant association between *Akkermansia muciniphila* and the expression of miR‐425‐3p. As miR‐425‐3p is associated with lipid metabolism, this study supports the concept that lipids are important players in microbial–epigenetic interactions (Tarallo et al. 2022).

Regarding dietary factors, tryptophan‐derived metabolites dysregulate miRNA expression and affect adiposity, insulin sensitivity, and energy balance (Virtue et al. 2019). Furthermore, dietary betaine may increase the abundance of SCFA‐producing bacteria and, thus, protect against obesity through SCFA‐mediated promoter methylation of obesity‐regulated genes via miR‐378a (Du et al. 2021). Finally, Assmann et al. (2020) demonstrated an association between specific miRNAs (miR‐130b‐3p, miR‐185‐5p, and miR‐21‐5p), involved in the regulation of metabolic pathways, and the abundance of *Bacteroides eggerthi*, potentially driving the progression of obesity.

In patients with depression, Chen et al. (2022) found a strong correlation between increased microbes and fecal miRNAs. Predicted miRNA functions include depression‐related pathways, circadian rhythms, and dopaminergic synapses. Specifically, the abundance of *Bacteroides* and *Dialister* was correlated with the expression of miR‐1278 and miR‐769‐3p in patients with depression. In addition, initial results suggested that microbiota‐induced increases in miR‐206‐3p expression in the brain tissue led to anxiety‐like behavior in mice (Q. Li et al. 2023). Using a mouse model, another study suggested a regulatory influence of the gut microbiota (*Lactobacillus and Alloprevotella*) on miR‐149 gene expression in the prefrontal cortex. The inhibition of miR‐149 reduced depression‐like behavior (Ma et al. 2022).

Although the involvement of miRNAs in the pathogenesis of AN has not yet been identified (Voelz et al. 2024), Schroeder et al. (2018) were the first to identify prenatally‐stress‐induced hypermethylation of miR‐340, which led to increased susceptibility to activity‐based anorexia (ABA) in the animal model. These findings support the hypothesis that other miRNAs may also play a role in AN.

### 1.6 Closing the Gap

Previous studies have demonstrated that gut microbiome alterations and epigenetic modifications occur in patients with AN and are likely relevant to the underlying pathophysiology (Käver et al. 2024; Garcia and Gutierrez 2023). However, the interactions between these mechanisms have not been investigated in AN. Research into AN still lacks a comprehensive framework that examines the overarching connections between individual changes. This is crucial to advancing our understanding of the pathophysiology of AN and addressing the limitations of current therapeutic approaches. The interplay between the gut microbiome and epigenetic factors has already been observed in other metabolic (obesity) and neuropsychiatric (depression) diseases (H. S. Lee 2019).

Insights into microbial–epigenetic interactions in AN may be informed by findings from these diseases. All three conditions show reduced microbial richness (Mörkl et al. 2017; Nikolova et al. 2021), suggesting that diversity loss may be relevant to creating a general interaction framework, potentially even more than specific taxa, given inconsistent community profiles across AN studies. In both obesity and AN, microbial shifts are primarily nutrition‐driven despite contrasting phenotypes; however, obesity features a *Firmicutes‐to‐Bacteroidetes* shift, enhancing energy extraction (Ramos‐Molina et al. 2019), while AN presents with enriched mucin‐degrading and pro‐inflammatory taxa, along with depleted butyrate‐producing and beneficial taxa as observed in depression (Zhao et al. 2024; Jiang et al. 2015). These alterations potentially contribute to intestinal permeability, low‐grade systemic inflammation, and reduced SCFAs, which may represent a core link between microbial and epigenetic changes in AN. SCFAs seem to be reduced across all three conditions, potentially mediating shared pathways (Sun et al. 2013; You et al. 2022). Epigenetic changes in overlapping genes related to appetite, energy metabolism, and stress, such as leptin/ghrelin pathways in AN and obesity and HPA axis regulation in AN and depression, further support a common framework (Käver et al. 2024; Kumar et al. 2014; Talarowska 2020). Despite condition‐specific contracts, these central parallels support the (partial) transferability of microbial–epigenetic interactions from obesity and depression to AN Table 1.

**TABLE 1:** Overview of selected shared and divergent microbial and epigenetic dysregulations in AN, obesity, and depression.

	AN	Obesity	Depression
Microbial alterations			
Reduced microbial richness	X (Ruusunen et al. 2019)	X (Mörkl et al. 2017)	X (Nikolova et al. 2021)
microbial shifts primarily nutrition‐driven	X (Zhao et al. 2024)	X (Hill et al. 2023)	
enriched mucine‐degrading taxa	X (Zhao et al. 2024)		
depleted butyrate‐producing taxa	X (Zhao et al. 2024)		X (Jiang et al. 2015)
*Firmicutes‐to‐Bacteroidetes* shift		X (Ramos‐Molina et al. 2019)	
Epigenetic alterations			
reduced SCFAs	X (Käver et al. 2024)	X (You et al. 2022)	X (Sun et al. 2013)
epigenetic changes in leptin/ghrelin pathways	X (Käver et al. 2024)	X (Kumar et al. 2014)	
epigenetic changes in HPA axis regulation	X (Kumar et al. 2014)		X (Talarowska 2020)
epigenetic changes in neuronal communication	X (Käver et al. 2024)		X (Yuan et al. 2023)
epigenetic changes in thermogenesis and lipid metabolism	X (Käver et al. 2024)	X (Kumar et al. 2014)	
epigenetic changes in weight regulation/metabolic pathways	X (Käver et al. 2024)	X (Kumar et al. 2014)	
restoration of epigenetic shifts within weight change	X (Käver et al. 2024)	X (Kumar et al. 2014)	

Changes in gut microbes and epigenetic factors are long‐lasting but often not permanent. Therefore, the interaction pathways between these players represent possible therapeutic targets (Begum et al. 2022; Yuan et al. 2023). Future longitudinal ABA animal studies, inducing typical changes in the microbiome and epigenetics, should perform systematic correlations of these two parameters. Thereafter, clinical observational studies should be conducted to indicate potential long‐term interactions between epigenetic changes and microbial dysregulation. Specific topics for future studies are developed below, based on existing literature as a foundation.

External stressors such as childhood trauma or social pressures may influence epigenetics and microbiota in AN, potentially contributing to lasting biological changes (Wang et al. 2024; Meneguzzo et al. 2022). For instance, reduced cortisol excretion and HPA‐hypoactivity have been observed in childhood trauma‐exposed patients with AN and could result from microbial and epigenetic alterations (Meneguzzo et al. 2022; Castellini et al. 2023). A longitudinal study assessing how different external stressors in AN jointly modulate epigenetics, microbiota, and clinical phenotypes might elucidate causal pathways linking environment, biology, and disease progression.

Also, the potential for developing predictive and diagnostic parameters based on microbial–epigenetic interactions should be evaluated in longitudinal clinical studies. The bacterial composition of the gut microbiome in patients with AN on hospital admission may predict hospital readmission. In particular, an increased abundance of *Sutterella* on admission has been correlated with increased body weight after 1 year (Andreani et al. 2024). Epigenetic alterations have been established as biomarkers for the early detection, diagnosis, and prognosis of cancer and may apply to psychiatric disorders (Neumann et al. 2020; Jankowska et al. 2015). Associating early epigenetic and microbial abnormalities with AN progression or longitudinal relapse in the same patient may reveal biomarkers. For instance, microbiome‐derived metabolites that correlate with epigenetic modifications could serve as early warning signs for disease onset and progression.

In a subsequent step, intervention studies should target possible therapeutic approaches.

Interestingly, epigenetic‐modifying medications, such as olanzapine, have already been recommended for AN therapy (Himmerich et al. 2023; Su et al. 2020; Guidotti et al. 2011). Few human and animal studies have investigated the therapeutic potential of fatty acid supplementation, probiotic supplementation, or FMT (Fan et al. 2023; Prochazkova et al. 2019; de Clercq et al. 2019; Keller et al. 2022; Navarro‐Tapia et al. 2021; Trinh et al. 2023; Gröbner et al. 2022; Wilson et al. 2023). In current studies, FMT of patients with AN in rodents appears to be feasible, and with simultaneous starvation, clinical parameters can also be mapped (Fan et al. 2023). Investigating the influence of FMT not only on microbial but also on epigenetic changes and the associations of these changes may be an interesting avenue of research. Previously, a study investigating systemic lupus erythematosus demonstrated the potential influence of FMT on epigenetic factors, as evidenced by increased *S*‐adenosyl‐l‐methionine and DNAm levels (B. Zhang et al. 2023). Moreover, therapies targeting the microbiome might be more effective when combined with strategies addressing specific epigenetic profiles, facilitating a more personalized and synergistic therapeutic approach. For instance, combining microbiome‐targeting treatments, such as probiotics or SCFA supplementation, with epigenetic reprogramming agents (e.g., sirtuin activators) could offer a promising dual approach to restoring normal metabolic and psychological functions in AN. Precisely, as deacetylases, sirtuins were found to improve fat mobilization, inflammatory processes, and insulin sensitivity in patients with obesity (Fraiz et al. 2021).

Altogether, initial evidence suggests that the interplay between gut microbiota and epigenetics may contribute to the pathophysiology in AN, beyond their established roles in obesity and depression. In AN, malnutrition and stress‐related hormonal changes may shape microbiota and epigenetics, which potentially influence each other. Current findings point to interactions mediated by microbial‐derived and epigenetically active SCFAs and correlations between microbial abundance and specific miRNAs. Systematic future research on this microbial–epigenetic interaction could advance understanding of AN pathophysiology and guide new treatment approaches.

## Author Contributions

**N.M. Korten**: conceptualization; data curation; writing – original draft. **A.C. Thelen**: conceptualization; data curation; writing – original draft. **C. Voelz**: conceptualization; funding acquisition; writing – review and editing. **C. Beyer**: funding acquisition; supervision; writing – review and editing. **J. Seitz**: conceptualization; supervision; writing – review and editing. **S. Trinh**: conceptualization; funding acquisition; writing – review and editing. **L. Käver**: conceptualization; data curation; supervision; writing – review and editing.

## Conflicts of Interest

The authors declare no conflicts of interest.

## Peer Review

The peer review history for this article is available at https://publons.com/publon/10.1002/brb3.70733

## Data Availability

Data sharing is not applicable to this article, as no new data were created or analyzed in this study.
